# Beyond decoration: free-standing lace embroidery for 3D shaped surgical mesh implants

**DOI:** 10.1038/s41598-026-36575-2

**Published:** 2026-03-04

**Authors:** Robert Tonndorf, Cindy Elschner, Alice Osterberg, Victor Mozer, Eduardo Antonio Wink de Menezes, Lilly Römer, Yordan Kyosev, Axel Spickenheuer, Christina Scheffler, Markus Stommel

**Affiliations:** 1 Division Polymer Materials Engineering, Leibniz Institute of Polymer Research Dresden, 01069 Dresden, Germany; 2 Chair of Development and Assembly of Textile Products, Institute of Textile Machinery and High Performance Material Technology, Dresden University of Technology, 01062 Dresden, Germany; 3 Faculty Design, University of Applied Sciences HTW Dresden, 01069 Dresden, Germany; 4 Institute of Construction Materials, Dresden University of Technology, 01062 Dresden, Germany; 5 Institute of Materials Science, Dresden University of Technology, 01062 Dresden, Germany

**Keywords:** Embroidery, Free-standing lace, Surgical mesh implant, Breast reconstruction, Textile engineering, Medical textiles, Engineering, Health care, Materials science, Medical research

## Abstract

Conventional mesh pockets manufactured from flat textiles by cutting and sewing often display wrinkles, excess material, and suboptimal fit on curved surfaces. This study demonstrates that free-standing lace embroidery enables the production of near-net-shape hemispherical mesh pockets with predefined pore geometries using standard embroidery machines. These pockets are scalable to different implant sizes and shapes. Experimental evaluations, including tensile and drop testing as well as 3D surface scanning, confirmed that the pockets are mechanically robust and exhibit excellent conformity with minimal wrinkling. These results highlight the potential of embroidery as a versatile platform for developing patient-specific surgical mesh implants.

**Supplementary Information:**

The online version contains supplementary material available at 10.1038/s41598-026-36575-2.

## Introduction

Machine embroidery is a textile technique in which pairs of threads are sewn onto a base material to create pre-programmed patterns^1^. Traditionally used in fashion for colorful, decorative designs, embroidery continues to attract attention in areas such as smart textiles^2–4^, and shape-changing fabrics^5^, and AI for accessible creation of patterns^6^. When performed on water- or chemically soluble substrates, it produces free-standing lace^7^, a structure held together solely by intersecting threads. Although free-standing lace has also long been used for decorative purposes, it has recently been explored for advanced uses, including fiber scaffolds for medical applications^8,9^, smart materials for the skin^10^, and auxetic structures^11^. A major advantage of embroidered structures is that their complete fiber architecture is predefined before manufacturing, enabling precise digital modeling and simulation^12^.

**Fig. 1:**
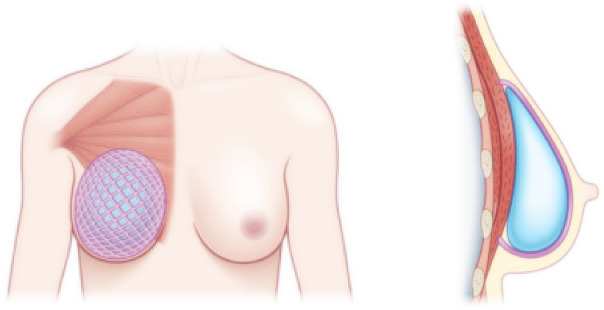
Schematic representation of the prepectoral implantation of a silicone implant with mesh shell in pink color. (adapted from^16^).

Surgical meshes are widely used in medicine to provide mechanical support and promote tissue integration. They are applied in procedures such as hernia repair, pelvic floor reconstruction, tendon reinforcement, and soft tissue augmentation^13^. These meshes are also used in breast reconstruction and augmentation to support soft tissue and maintain breast implant positioning. Breast implants are used for both augmentation and post-mastectomy reconstruction. Implants can be placed either subpectorally (beneath the chest muscle) or prepectorally (above the muscle) (Fig. 1). Prepectoral placement generally results in less pain and improved postoperative mobility for the patient^14,15^. To reinforce the surrounding tissue and prevent implant displacement, biological meshes (acellular dermal matrices, ADM) and synthetic absorbable (Vicryl^®^), long-term absorbable (TIGR^®^), or nonabsorbable titanium-coated polypropylene (TiLOOP^®^) woven and knitted textile meshes are increasingly used to form supportive shells around silicone implants^16–18^. Although these flat meshes are primarily intended for submuscular reconstruction, they are also used in prepectoral breast reconstruction, where surgeons manually fold and suture them into a pocket shape during surgery to fit the implant^19–21^.

However, fabricating a pocket from a flat mesh intraoperatively increases operative time and may lead to suboptimal fit or implant migration. To reduce operative time, commercially available prefabricated pockets can be used, such as the non-absorbable TiLOOP^®^ Bra Pocket (mass per unit area 16 g/m²; marketed by PFM Medical Titanium GmbH^22^, and the absorbable SERASYNTH^®^ MESH Pocket (mass per unit area 90 g/m², marketed by Serag-Wiessner^23^, and the pre-shaped ADM Braxon (0.6-mm-thickness, marketed by Medical Biomaterial Products (mbp) GmbH, Neustadt-Glewe, Germany^24^.

Although intraoperatively shaped and prefabricated pockets provide a close fit to the implant, improvements can still be made to the exactness of the fit and the efficiency of the material used. Flat textiles cannot be draped evenly over a hemispherical implant, so folds develop in these pockets, reducing three-dimensional conformity. Furthermore, the conventional folding and sewing process creates localized thickening at the seams where the mesh layers overlap.

This study investigates the use of free-standing lace embroidery to produce highly customized textile meshes. As a complex, proof-of-concept study, the design and development of a mesh pocket for breast augmentation are presented. The embroidery pattern design workflow and its translation into a functional surgical mesh are detailed, highlighting the precision and design freedom achievable using only standard flat-bed or cylinder-type embroidery machines and thread.

## Methods

### Silicone implant model

**Fig. 2:**
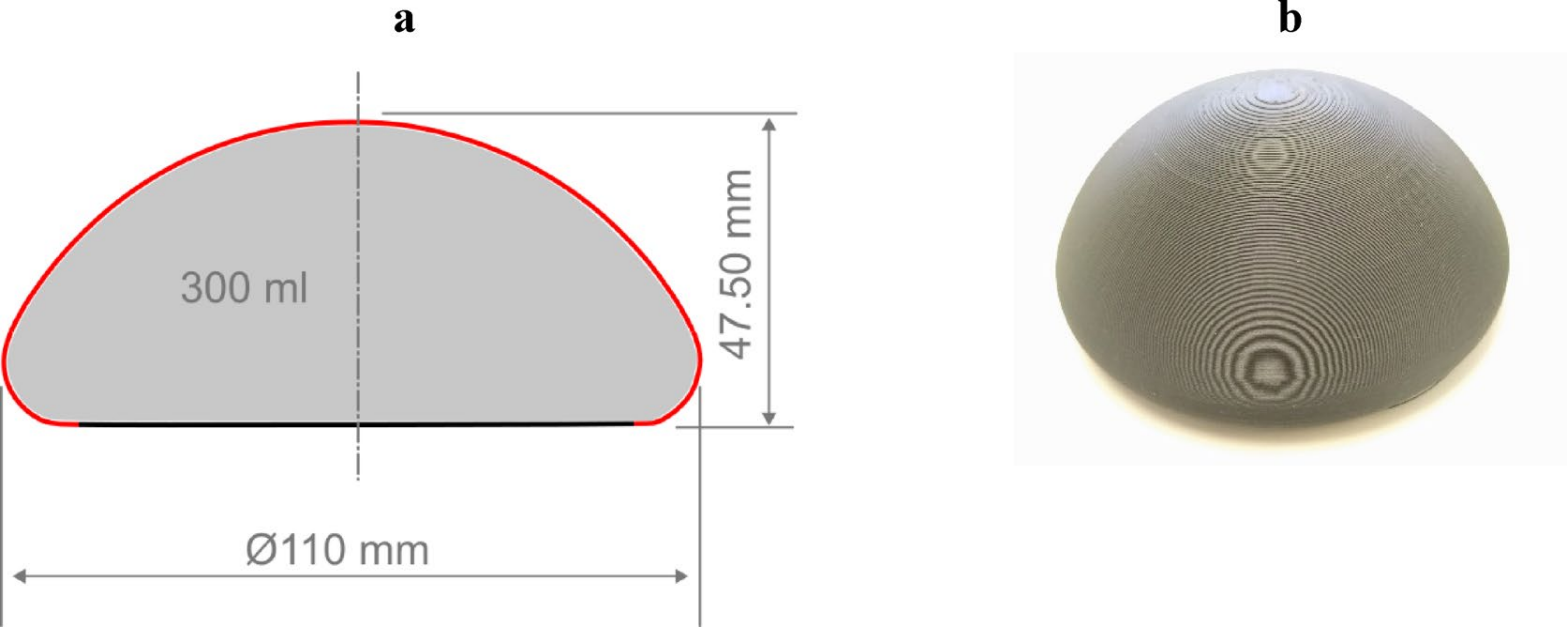
**a** Cross-sectional scheme of the 3D-printed model, marked in red indicates the surface of the implant to be covered by the mesh cover; **b** photo of the 3D-printed silicone implant model made from PLA without the silicone cover.

The mesh pocket is designed to cover a silicone implant with defined dimensions. For this study, a 3D-printed model of a silicone implant measuring 47.50 mm in height, 110 mm in diameter, and 300 ml in volume was used (Fig. 2a). For the larger mesh, a corresponding implant model with a volume of 600 ml, a height of 59.80 mm, and a diameter of 139 mm was employed. The models were printed using a Bambu Lab X1-Carbon 3D printer with a polylactide (PLA) filament (Fig. 2b). Additionally, a silicone cover was created by casting and curing 2 K silicone (Silicone Rubber ADDV-42 from R&G Faserverbundwerkstoffe GmbH) over the 3D-printed model, resulting in a casing that accurately fits the implant model. During mechanical testing, this cover was used to ensure a smooth and even surface on top of the staircase-like 3D-printed surface.

### Embroidery pattern

**Fig. 3:**
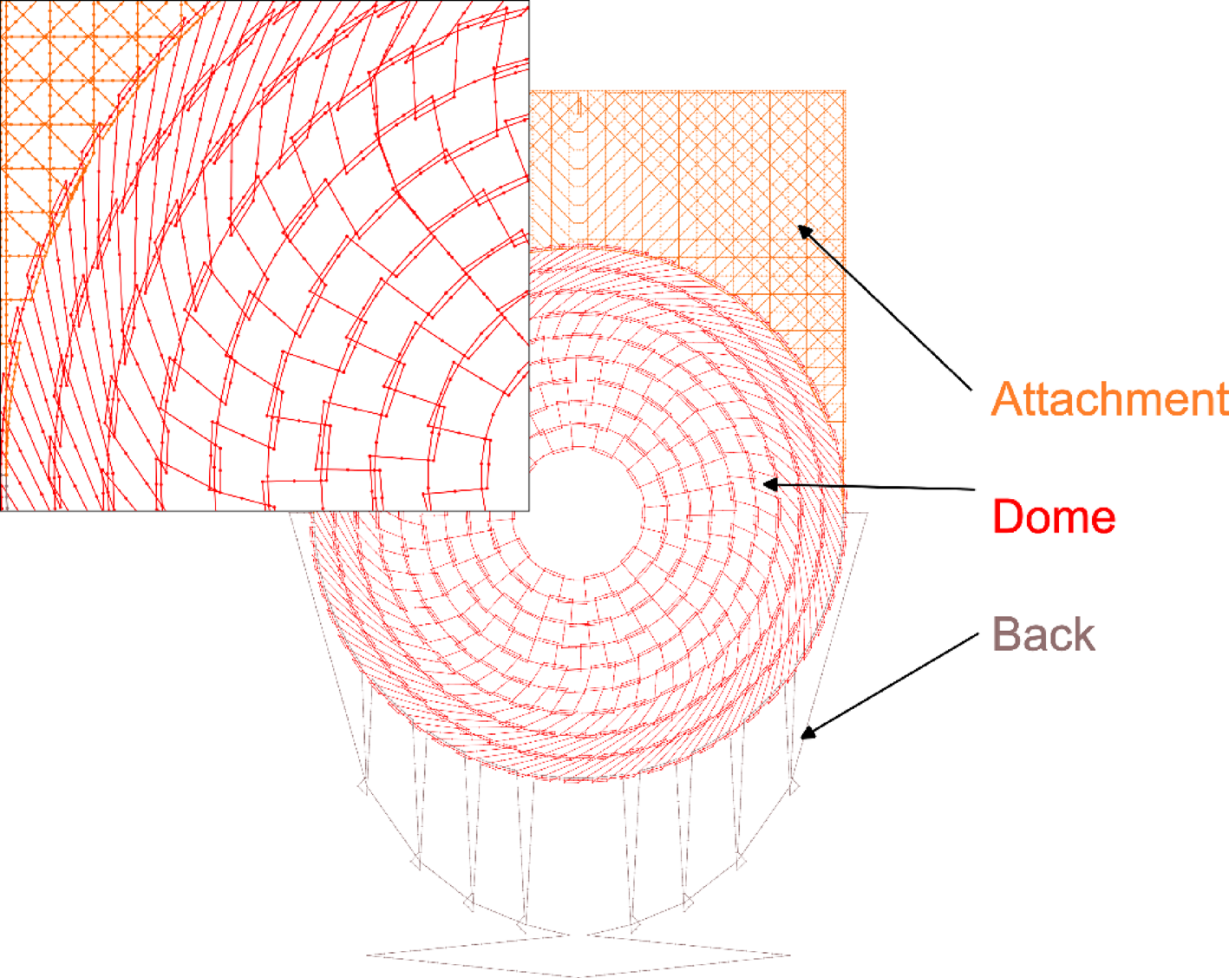
Overview and enlarged inset of the embroidered pattern, consisting of three components: the dome, the back, and the attachment. The angled, meandering paths between the dome’s concentric rings act as thread reservoirs. When tensioned during draping, the rings rotate relative to each other, converting the flat embroidery into a hemispherical form. Stitches are indicated by dots.

The parametric, constraints-based 2D-Sketch function in SolidWorks 2023 was used to design the embroidery pattern (Fig. 3). The mesh pocket consists of three components: the dome, the back, and the attachment. The dome is the functional part that reinforces the surrounding biological tissue. The back holds the implant in place inside the pocket. The attachment connects to the dome and fixes the mesh pocket in a specific location inside the body.

The dome consisted of several concentric rings, with neighboring rings connected by meandering paths. The geometry of these meandering paths changed from inside to outside of the dome, with the connection lines for the outer rings becoming increasingly angled. The back side was constructed from multiple loops connected to the lower part of the dome, with a single path running through them that connected the left and right sides of the dome. The attachment consisted of two layers of parallel paths at 0° and 90°, as well as an additional diagonal layer at ± 45° near the outer corners.

### Embroidery pattern variants

**Fig. 4:**
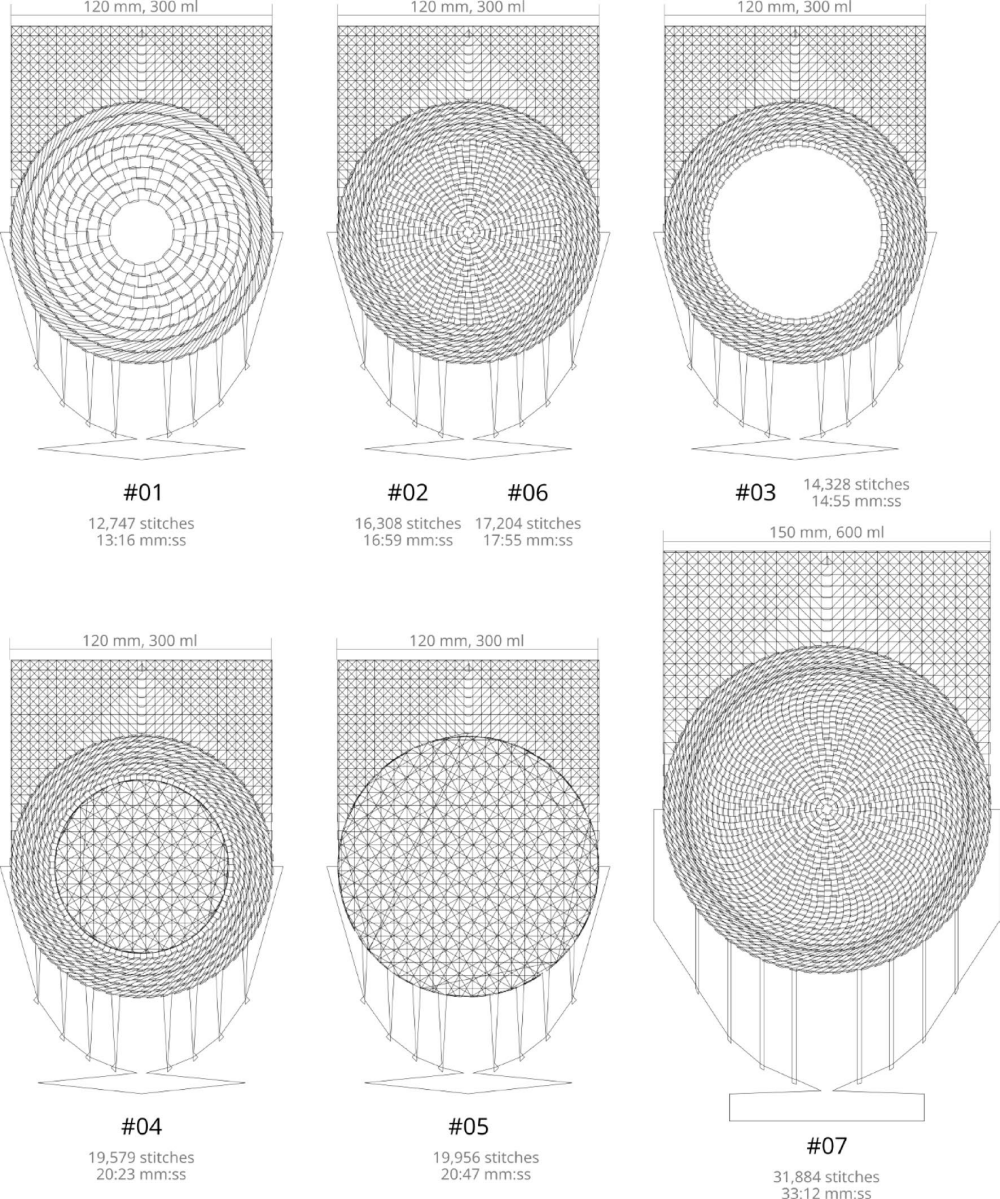
Mesh cover designs – **#01** standard; (**#02** = **#06**) narrower ring spacing; (**#03**) truncated cone with open center; (**#04**) truncated cone with flat center through crossed paths; (**#05**) flat crossed-path pattern; (**#07**) enlarged (based on #02 and #06) for a 600 ml implant.

To systematically investigate how specific design parameters affect fit, mechanical performance, and pore architecture, different embroidery patterns were developed. These parameters included spacing between concentric rings, dome closure, additional reinforcement threads, and upscaling. A standard mesh coverage pattern (**#01**) was established and several alternative designs were derived to explore these variables (Fig. 4). Pattern **#02** is similar to the standard design, but with narrower spacing between the concentric rings. Pattern **#03** features a truncated cone shape with a large open center. Pattern **#04** also has a truncated cone shape, but with a flat top instead of an opening. It uses a simple crossed-path pattern instead of concentric rings and meanders. Pattern **#05** is completely flat and lacks concentric rings and meanders, employing a simple crossed-path design instead. Pattern **#06** modifies pattern **#02** by adding a threefold stitching path running through the loops on the back for reinforcement. All of these mesh pockets were tailored for a 300 ml implant. Pattern **#07** is similar to **#02** but has been scaled up for a 600 ml implant.

### Machine embroidery

All embroidery patterns were designed as a single continuous stitch path, allowing the mesh pockets to be fabricated in one step. This approach eliminates the need for thread trimming or jump stitches, which would otherwise produce open ends and loose filaments. Patterns were exported from SolidWorks as a DXF file and converted into a DST file readable by any embroidery machine using the Ink/Stitch plugin (version 3.0.1) for Inkscape (version 1.3.2).

Embroidery was performed using a flat-bed Tajima TLMX-901 embroidery machine (Tajima Group, Japan). Polypropylene (PP) monofilaments (G. Krahmer GmbH, Germany) with diameters of 150 μm, 125 μm, 67 μm, and 49 μm, respectively, were used for both the top and bottom threads. The standard pattern (#01) was produced with all filament types, while all other patterns were embroidered using 125 μm filaments. A polyvinyl alcohol (PVA) nonwoven substrate (DO 105, Freudenberg Performance Materials Apparel GmbH) was used as a temporary base material for stitching. The stitching speed was set to 1000 stitches per minute. Depending on the pattern, the embroidery duration ranged from approximately 13 to 21 min for the 300-ml implant pockets and around 33 min for the pocket designed to fit a 600-ml implant. After completion, the embroidered pattern became self-supporting due to the intersecting stitch paths. The PVA substrate was then dissolved in water overnight, resulting in a free-standing thread structure.

### Dome shaping and pocket closure

The free-standing lace structures were draped over a hemispherical 3D-printed implant model. This shaping was achieved without fold formation due to the specific design of the connecting segments between the rings. These segments were embroidered in a meandering (zig-zag) pattern, acting as “thread reservoirs”. During the shaping process, these reservoirs allowed the concentric rings to rotate and shift relative to one another, as the stored filament material was released, preventing tension on neighboring rings. This mechanism converted the initially flat embroidery into a hemispherical form without the material bunching or wrinkling, with the connecting threads eventually aligning perpendicular to the rings.

Finally, the thread corresponding to the back-side segment of the pattern was manually pulled through the loops. These loops, connected to the lower part of the dome, were folded to the back side of the 3D-printed implant model, and the thread was tightened and knotted, resulting in a closed back side of the pocket.

### Estimation of the shape of the dome

**Fig. 5:**
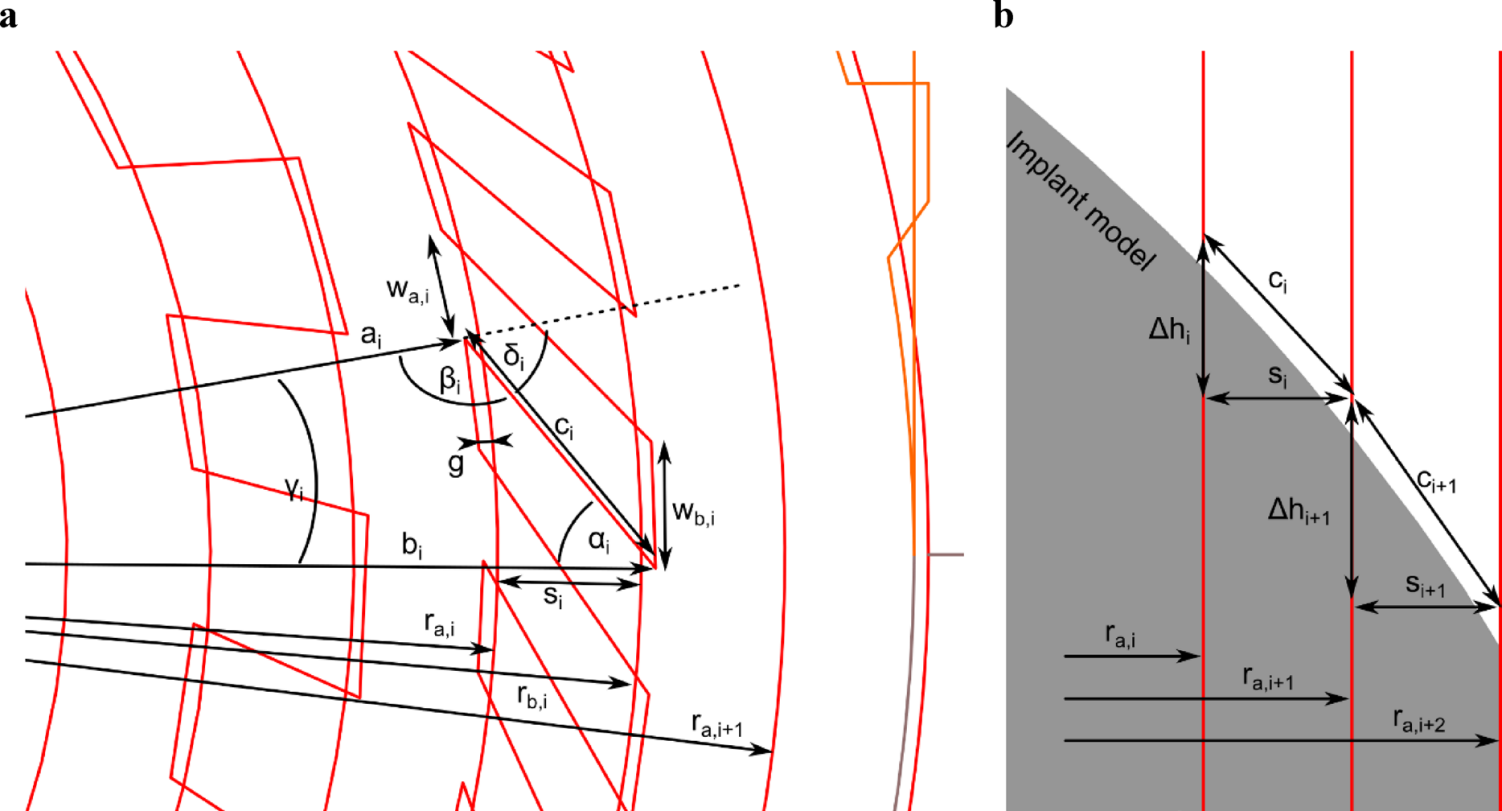
**a** Top-view of the embroidered pattern. **b** Detailed side-view showing 2 meandering paths with differing geometries between adjacent concentric rings, with stitch points indicated; remaining meandering paths are omitted for clarity.

To predict how the flat 2D pattern would transform into a 3D dome, the length of the connecting threads, which act as thread reservoirs, was calculated using trigonometric relationships. The goal was to determine the exact height difference between the rings once the mesh was expanded, thereby defining the shape of the expected mesh curve. All relevant variables for the calculation are as follows:

- $\:{r}_{a,i}$, $\:{r}_{b,i}$: Radii of the inner and outer concentric rings, respectively.
- $\:{s}_{i}$: Radial spacing between two adjacent rings.
- $\:g$: Overlap distance at the connection points between rings.
- $\:{a}_{i}$, $\:{b}_{i}$: Adjusted radii for inner and outer rings, accounting for the overlap distance $\:g\:$.
- $\:{c}_{i}$: Length of the connecting (meandering) path between two adjacent rings.
- $\:{\delta\:}_{i}$: Angle used for pattern construction in CAD.
- $\:{\alpha\:}_{i}$, $\:{\beta\:}_{i}$, and $\:{\gamma\:}_{i}$: The three interior angles of the triangle formed by the adjusted radii ($\:{a}_{i}$ and $\:{b}_{i}$) and the connecting path ($\:{c}_{i}$).
- $\:{\varDelta\:h}_{i}$: Maximum achievable vertical height difference between two adjacent rings after draping.
- $\:{w}_{a,i}$, $\:{w}_{b,i}$: Circumferential spacing between adjacent connecting paths on the inner and outer rings.

The length $\:{c}_{i}$ was calculated using the law of sines (Eq. 1), the sum of angles (Eq. 2), and the law of cosines (Eq. 3). By taking into account the diameters of the two concentric rings and the overlap distance $\:g$ (Eq. 4 and Eq. 5) and with an angular relationship (Eq. 6), a trigonometric relationship between the pattern parameters and the resulting dome shape could be calculated (Fig. 5a).

1 $$\:\mathrm{sin}{\alpha\:}_{i}=\frac{{a}_{i}}{{b}_{i}}\bullet\:\mathrm{sin}{\beta\:}_{i}$$

2 $$\:180={\alpha\:}_{i}+{\beta\:}_{i}+{\gamma\:}_{i}$$

3 $$\:{c}_{i}^{2}={a}_{i}^{2}-2\bullet\:{a}_{i}\bullet\:{b}_{i}\bullet\:\mathrm{cos}{\gamma\:}_{i}+{b}_{i}^{2}$$

4 $$\:{a}_{i}=\:{r}_{a,i}\:-\:g$$

5 $$\:{b}_{i}=\:{r}_{b,i}+\:g$$

6 $$\:{\beta\:}_{i}=180-{\delta\:}_{i}$$

By rearranging and substituting into the equations, the connection path length $\:{c}_{i}$ could be determined (Eq. 7, Eq. 8 and Eq. 9).

7 $$\:{\alpha\:}_{i}={\mathrm{sin}}^{-1}(\frac{{a}_{i}}{{b}_{i}}\bullet\:\mathrm{sin}{\beta\:}_{i})$$

8 $$\:{\gamma\:}_{i}={-\beta\:}_{i}-{\alpha\:}_{i}+180$$

9 $$\:{c}_{i}=\sqrt{{a}_{i}^{2}-2\bullet\:{a}_{i}\bullet\:{b}_{i}\bullet\:\mathrm{cos}{\gamma\:}_{i}+{b}_{i}^{2}}$$

After draping the pattern around the implant model, and assuming that all connecting paths between the rings were fully tensioned and perpendicular to the concentric rings, trigonometric calculations were applied to estimate the resulting dome shape (Fig. 5b). By applying the Pythagorean theorem (Eq. 10) and considering the radial distance $\:{s}_{i}$ between the rings (Eq. 11) the maximum achievable height difference $\:{\varDelta\:h}_{i}\:$ between adjacent rings could then be calculated (Eq. 12).

10 $$\:{c}_{i}^{2}=\varDelta\:{h}_{i}^{2}+{s}_{i}^{2}$$

11 $$\:{s}_{i}={r}_{b,i}-{r}_{a,i}$$

12 $$\:{\varDelta\:h}_{i}=\sqrt{{c}_{i}^{2}-{s}_{i}^{2}}$$

### Pore size calculation

After draping and shaping, the pores within the dome region can be approximated by isosceles trapezoids. The bases of these trapezoids lie on two neighboring concentric rings, with the longer base on the outer concentric ring and the shorter base on the inner concentric ring. The base lengths ($\:{w}_{a,i}$ and $\:{w}_{b,i}$) were determined by the circumferential spacing between adjacent connecting paths on the respective rings, as specified by the stitch pattern (Fig. 5a). Pore width was defined as the mean of the two base lengths (Eq. 13). The legs were set by the length of the connecting paths $\:{c}_{i}$ (Eq. 9), which link two concentric rings. As all connecting paths in one radius of the pattern are set with the same angle $\:{\delta\:}_{i}$ it follows that both legs of the trapezoidal pore have the same length. The pore height was calculated from $\:{w}_{a,i}$, $\:{w}_{b,i}$, and $\:{c}_{i}$ applying the Pythagorean theorem (Eq. 14). Pore size dimensions $\:w$ and $\:t$ were then analyzed and plotted for the patterns with a dome shape: #01, #02 (equivalent to #06), and #07.

13 $$\:{w}_{i}=\frac{{w}_{a,i}+{w}_{b,i}}{2}$$

14 $$\:{t}_{i}=\sqrt{{c}_{i}^{2}-{\left(\frac{{w}_{b,i}+{w}_{a,i}}{2}\right)}^{2}}$$

### Mechanical characterization

#### Filament tensile testing

The different filaments used for embroidery were mechanically characterized by uniaxial tensile testing to determine their tensile force at break. This procedure aligns with standard practice for monofilament evaluation (DIN EN ISO 2062), employing a zwickiLine testing machine. Results are reported in cN/tex (centinewtons per tex), a standard unit of tenacity in textile engineering. This value represents the strength-to-weight ratio of the fiber, describing the breaking force relative to the linear density (thickness) of the filament. Using this metric allows for a direct comparison of material strength independent of the filament’s diameter. Ten samples were tested for each filament type with a load cell of 10 N and a sample length of 250 mm, subjected to a preload of 0.5 cN/tex. The test speed was set to 250 mm/min; a rate commonly employed for similar monofilament materials within the ISO 2062 framework to ensure consistent data acquisition and comparability with established literature.

#### Mass per unit area determination

The mass per unit area (g/m²) was determined specifically for the embroidered dome segment to allow for the normalization of mechanical testing data. This approach was adopted because the dome structure was the only element systematically varied across the different patterns. For each pattern, the mass of the isolated dome segment was measured using a precision scale. This mass was then divided by the corresponding surface area of the 3D-printed implant model’s dome (indicated in red in Fig. 2a) that the segment was intended to cover. By focusing exclusively on the functional dome area and excluding contributions from other potential mesh components or the initial flat embroidery pattern, this method yields a relevant and comparable metric for evaluating the mechanical properties of mesh samples with varying dome designs.

#### Mesh pocket tensile testing

**Fig. 6:**
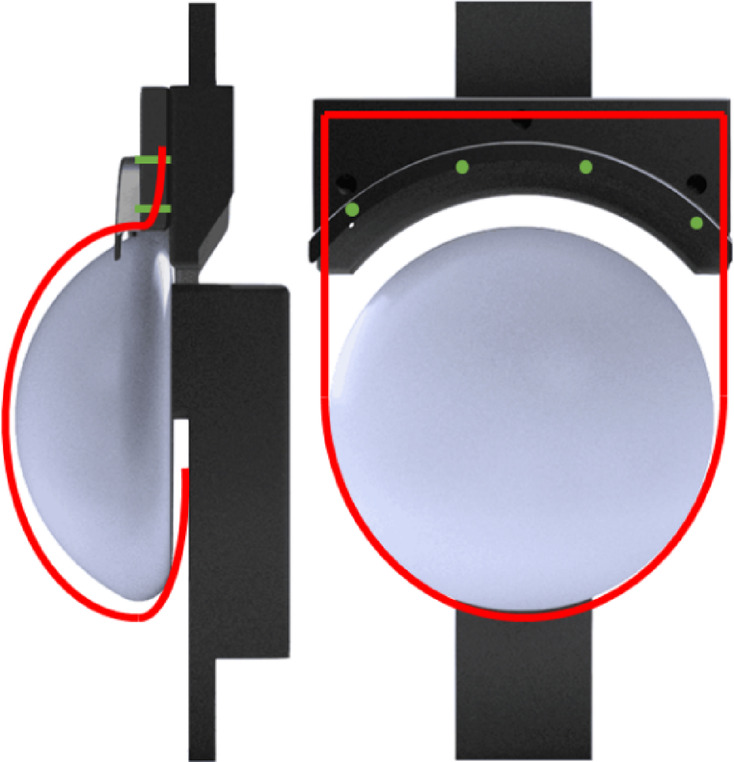
CAD model of the custom-built test rig replicating prepectoral pocket fixation, with the contour sketch (red line) indicating the mesh pocket position and green lines indicating the fixation points.

Mechanical testing of the embroidered mesh pockets was performed using a custom-built test rig designed to replicate prepectoral pocket fixation conditions (Fig. 6). As no standardized test framework exists or is described in academic literature for these specific mesh pockets, the rig was developed with considerations for workflow efficiency, ease of handling, and minimization of potential error sources.

A 3D-printed model implant with a defined shape and volume (i.e., 300 ml and 600 ml) was covered with the casted silicone cover, described in 2.1, to ensure a smooth surface independent of the 3D printed texture. The model implant was connected on its back side via struts to the lower clamps of a zwickiLine testing machine. Concurrently, the upper clamps were equipped with a frame featuring four mounting pins as fixation points. Prior to testing, the mesh pocket was positioned by pulling it up over the model implant from below and attaching it to the four fixation points. Subsequently, the test was initiated, causing displacement between the lower and upper clamps at a speed of 100 mm/min, utilizing a 10 kN load cell and a 0.5 N preload. This test speed was selected to allow detailed observation of force-displacement behavior and failure modes while ensuring reasonable test duration. Ten samples were tested for each pattern dome type.

Statistical analysis was performed to evaluate the differences in maximum tensile force at break between the different mesh patterns and filament diameters. Data are presented as mean ± standard deviation. A one-way analysis of variance (ANOVA) was conducted to determine the overall significance of differences between groups. Post-hoc pairwise comparisons were performed using the Tukey HSD (Honestly Significant Difference) test to identify specific differences between groups. A probability value (p-value) of < 0.05 was considered statistically significant.

#### Impact testing (drop tests)

Impact tests were performed to assess the resistance of mesh pockets to sudden mechanical loads that may occur in real-life scenarios (e.g., accidental impacts, falls, or physical activities). Since standardized impact tests for surgical meshes simulating implant containment are not established, the drop height was selected based on related literature. According to recent sports science research, the upper safe limit for plyometric drop jumps in trained athletes is 500 mm^25^. Thus, 500 mm was chosen as the reference height. The implant was placed inside the pocket, utilizing either a 3D-printed model (mass: 98 g) or a real silicone implant (mass: 277 g). This assembly was suspended by its four fixation points from a carriage frame guided by two vertical rails, oriented with the dome hanging downwards. The frame was released from a height of 500 mm and fell along the rails with minimal friction. The free fall was terminated abruptly by steel bolts inserted into the rails, which acted as a rigid mechanical stop. Upon impact, the frame stopped instantaneously, while the inertia of the enclosed implant exerted a dynamic tensile load on the mesh pocket. The integrity of the pocket and the containment of the implant were then assessed. Success was defined as the implant remaining securely within the pocket without displacement or mesh damage. Failures included the implant falling out or significant mesh breakage.

### Finite element analysis

Finite Element Analysis (FEA) was employed to simulate the uniaxial tensile loading scenario of the mesh pocket. The primary objectives of performing FEA were to complement the experimental mechanical testing by visualizing stress distribution within the mesh pocket, thereby identifying critical regions of stress concentration and deriving potential design improvements to enhance mechanical stability based on these findings. The simulation was conducted using ANSYS 2024 R1. The geometries of the implant and the pocket were imported from SolidWorks. As the stresses in the implant were not relevant, it was modeled as a rigid body. The pocket material (the attachment, dome, and back) was defined as a linear, isotropic material with a Young’s modulus of E = 5,600 MPa and a Poisson’s ratio of ν = 0.35, with a uniform embroidery pattern and constant thickness of 1 mm. Due to symmetry along the YZ plane, only half of the model was simulated (Suppl. Figure 1). The implant was meshed with a 10 mm element size and refined to 1.5 mm at the faces in contact with the pocket. To eliminate the bending stiffness of the embroidered structure, the pocket was modeled using linear membrane elements, generally set to 1.5 mm, and refined to 0.5 mm in regions where stress concentrations were expected. Contact between the pocket and the implant was modeled using the augmented Lagrange method with a friction coefficient of 0.3. The boundary conditions included fixing the implant at its two bolt holes and imposing a − Z displacement of 10 mm on the pocket attachment. Symmetry constraints were applied to the cut edge of the pocket.

### 3D surface scanning

3D surface scans were performed to evaluate the fit of the embroidered mesh pockets around the implant models, and to identify any folds or wrinkles. To enable a rapid assessment, both the implant and the mesh covers were scanned using the 3D-Scanner Artec Leo. It can capture small, highly detailed static objects without any contact between the device and the object being recorded. The linear field at closest range is 244 × 142 mm and 838 × 488 mm at furthest range. Consisting of a 3D camera and a color camera, the scanner can capture both geometry and textures with a 3D-resolution of up to 0.2 mm and a texture resolution of 2.3 megapixels. The resulting polygon model can be exported in various file formats, such as *.ply and *.obj. To reduce reflections and improve surface recognition, a thin, washable matting layer was sprayed onto the samples. This makes the surface of the object to be scanned white, bright and matt, while ensuring that all details and subtleties remain visible. Dry shampoo was used for this purpose. This mattifies the surface and improves scan results. The mesh fit was analyzed using cross-sectional evaluation. First, the digital 3D-printed implant model and the embroidered mesh were aligned in Blender through translation and rotation and exported as a *.ply file. This file was then processed in ParaView, where cross-sections were generated using the “Slice Datasets with Planes” function. The gaps between the resulting contour lines of the implant model and the mesh were color-filled to visualize the distance between both surfaces.

A second scanning setup was conducted for a more detailed analysis. This setup used an ATOS Q scanner with interchangeable lenses that could adjust the resolution and field of view. The camera and projector lenses were pre-calibrated for a measurement volume of 170 × 130 × 130 mm³, which enabled high precision with a moderate number of scans. Reference markers with a diameter of 0.8 mm were placed directly on the standard mesh sample (#01) and the implant model to enable accurate alignment of multiple scans. To minimize reflections, a removable scanning spray was applied to the surfaces of the mesh and implant. First, the implant model was scanned alone and fixed in position with a support stick to serve as the reference body. Then the mesh pocket was draped around the implant model in the same position as before and rescanned. The resulting datasets were aligned and any displaced scans were corrected by matching reference points on the reference body. The combined scans were then polygonized and the reference body was transformed into a computer-aided design (CAD) file format to enable further evaluation of the mesh fit and surface conformity by calculating the distance between the sample and the reference body.

## Results

### Embroidery pattern variants

Several embroidery patterns were successfully fabricated. These included designs based on concentric rings connected by meandering paths, which were intended to form closed dome structures (patterns #01, #02, #06, and #07) as well as an open dome structure (#03). In addition, patterns with a flat central segment instead of a dome were developed (#04 and #05).

**Fig. 7:**
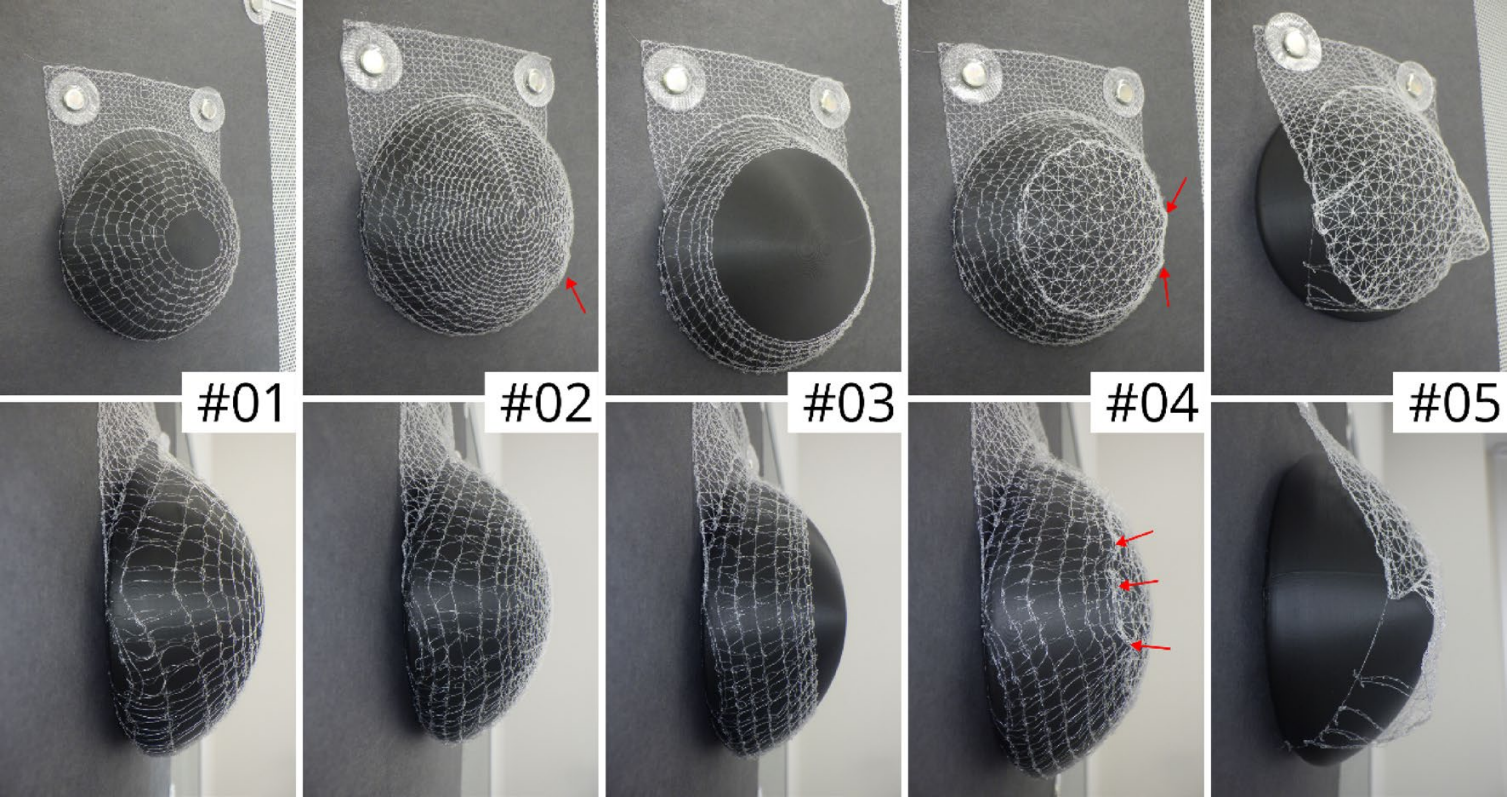
Embroidered mesh pockets with inserted 3D-printed implant model. Patterns #01 - #03 provided stable coverage, #02, and #04 showed minor folds in the dome region indicated by red arrows, and #05 failed to form a cavity to hold the implant.

The embroidered mesh pockets allowed the 3D-printed implant model to be inserted smoothly (Fig. 7). Patterns #01 through #03 provided stable fixation of the implant. Minor folds were observed in the dome region of patterns #02 and #04. For pattern #04, these folds were more pronounced, attributed to the intentionally flat central segment; however, these subtle imperfections were difficult to visualize clearly in standard photographs, underscoring the value of the 3D surface scans for detailed assessment of fit. In contrast, pattern #05, with its completely flat design lacking any cavity-forming structure, failed to hold the implant.

### Estimation of the shape of the dome

The calculations for estimating the dome shape revealed a direct relationship between the geometry of the meandering connection paths and the resulting curvature of the dome, which also explains the minor folds observed in pattern #02. A pyramidal shape was inevitable, even when the angle $\:{\delta}_{\mathrm{i}}$ of the meander’s connecting segments - controlling the dome curvature - was low or set to zero. This is because the connecting paths are positioned with a defined offset that overlaps with the distance $\:g$ between the concentric rings. This offset is required to ensure continuity between the meander path and the rings; if $\:g$ is too small, the paths fail to overlap, leading to discontinuities or holes in the mesh. However, the offset creates an unintended thread reservoir, which promotes pyramidal dome formation when the structure is stretched outwards, as all the gaps between the concentric rings expand by the same radial distance.

15 $$\:{c}_{i}^{*}={c}_{i}\bullet\:k$$

16 $$\:{\varDelta\:h}_{i}=\sqrt{{{c}^{*}}_{i}^{2}-{s}_{i}^{2}}$$

**Fig. 8:**
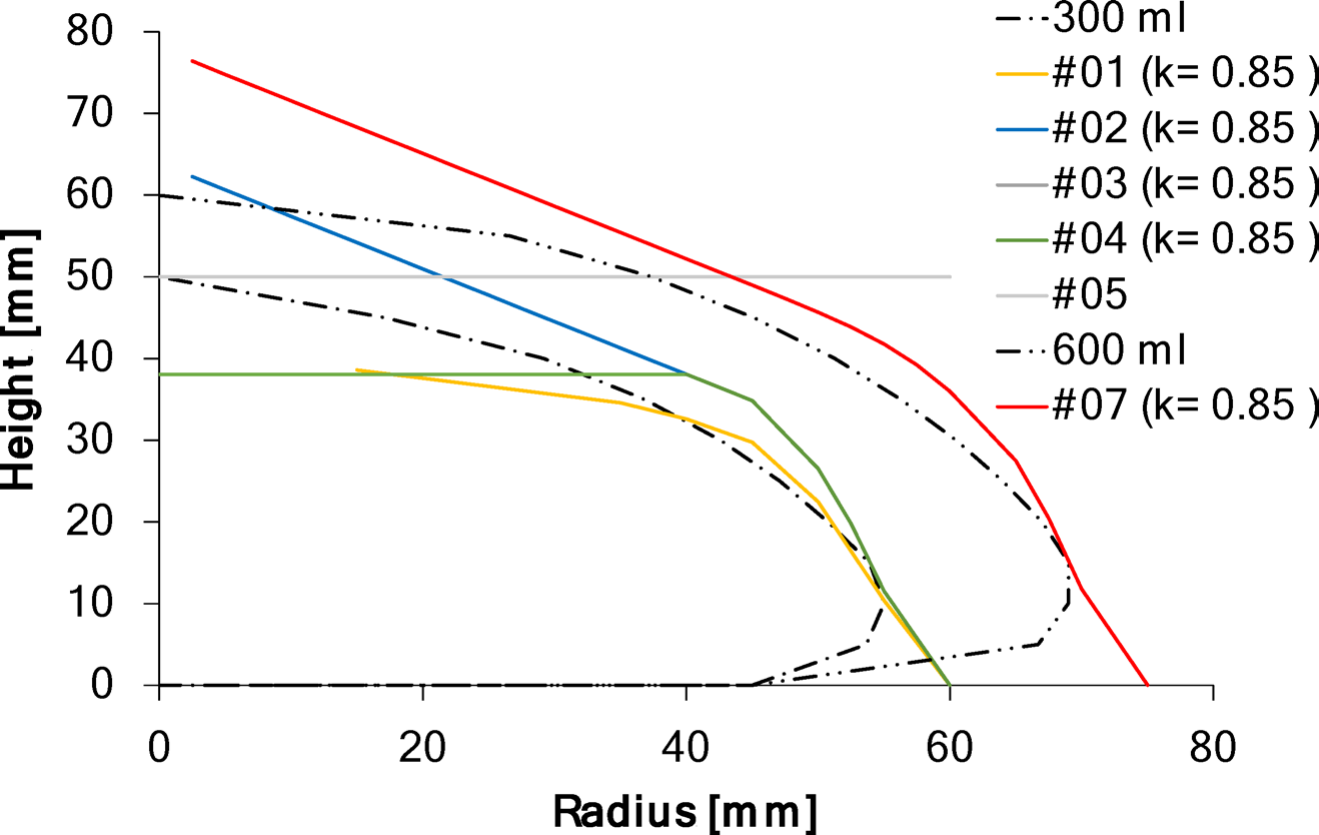
Calculated dome shape based on geometric parameters and with consideration of a correction factor (k ≈ 0.85); solid lines correspond to the shape of the implant model.

Additionally, it was observed that the height and curvature of all mesh domes were lower than predicted by the preliminary calculations. This deviation was attributed to the stiffness of the monofilament. When the stabilizing substrate is dissolved, the filament tends to spring back towards its original shape, resulting in curved and shortened filament segments between stitches. These elastic restoring forces counteract the intended expansion, effectively shortening the connecting paths and reducing the overall dome curvature. Introducing the empirical correction factor $\:k\:\approx\:\:0.85$ accounted for this effect, bringing the predicted mesh geometry closer to the experimental observations (Fig. 8).

### Pore size calculation

**Fig. 9:**
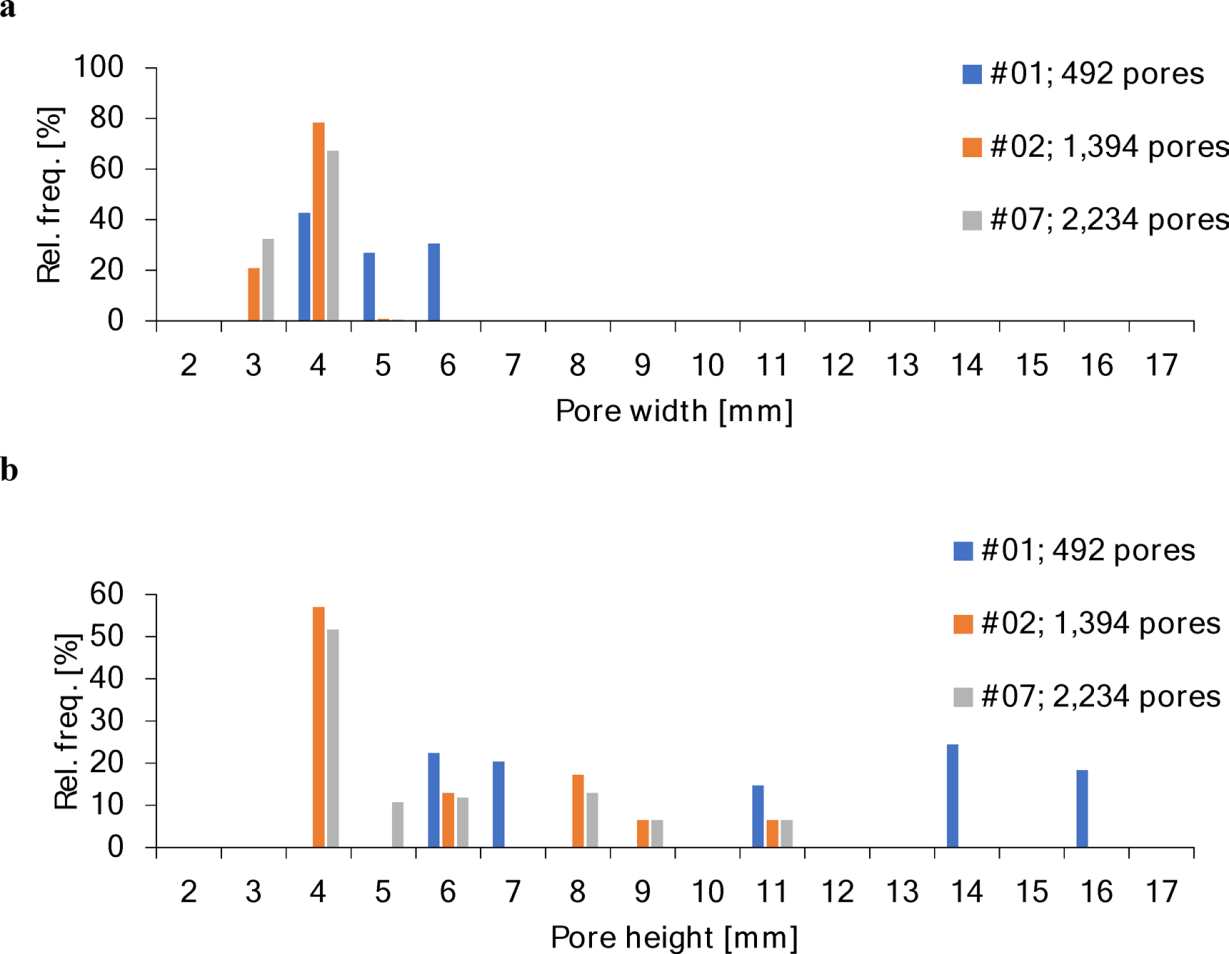
Pore size distribution plots of **a** pore width and **b** pore height (relative frequency) for patterns #01, #02 (= #06), and #07.

Based on the calculations and applying the correction factor (k ≈ 0.85), the pore size distribution of the dome was determined (Fig. 9). Pores were defined as trapezoidal and, thus, characterized by a width $\:w$ and a height $\:t$. The standard pattern #01 contained 492 pores, pattern #02 (=#06) with narrower ring spacing contained 1,394 pores, and the large mesh pocket with the same narrow ring configuration contained 2,234 pores. For patterns #01, #02 (=#06), and #07, the median pore width was 4 mm. The median pore height for patterns #02 (=#06) and #07 was also 4 mm, whereas pore height varied significantly for pattern #01, ranging evenly between 6 mm and 16 mm. All three embroidery patterns were constructed from concentric rings with uniformly increasing diameters, while the connecting paths defining the pore heights gradually increased in angle toward the outer region. Thus, this approach resulted in greater pore heights in these outer areas. The effect was particularly pronounced in pattern #01, since the distance between consecutive concentric rings in its stitch pattern was twice that of patterns #02, #06, and #07.

### Mass per unit area

The mass per unit area is defined as the mass of the dome relative to the surface area of the implant, considering only the dome surface of the implant and not the actual mesh surface (i.e., holes in the mesh were ignored). It was used to normalize mechanical test data of the mesh pockets. The total mesh mass depends on the diameter of the filament, as well as the size and geometry of the mesh. As expected, a clear decrease in the dome’s mass per unit area was observed as the filament diameter decreased, ranging from 15 g/m² for 150 μm filaments to 2 g/m² for 49 μm filaments for the standard pattern (#01). Increasing the mesh density by using 125 μm filaments results in a mass per unit area increase from 12 g/m² for pattern #01 to 23 g/m² for pattern #02. Enlarging the central circular opening reduced the mass per unit area to 14 g/m², as less filament material covered the implant surface (pattern #03). For the pattern with a flat central part (#04), an increased mass per unit area of 31 g/m² was determined. Scaling the mesh pocket from 300 ml to 600 ml did not significantly affect the dome segment’s mass per unit area, as this enlargement was achieved by increasing the number of concentric rings and corresponding meander structures.

### Mechanical characterization

The tensile strength of the filaments was used to normalize the maximum tensile force of the domes, enabling comparison between different dome patterns. The mean tensile strengths of the filaments used for mesh fabrication were 56.3 ± 1.3 cN/tex (diameter 49 μm), 58.9 ± 0.4 cN/tex (diameter 67 μm), 61.0 ± 0.5 cN/tex (diameter 125 μm), and 76.9 ± 1.3 cN/tex (diameter 150 μm). These values were used to normalize the results of the tensile tests of the pattern variants. For comparison, the tensile force of pattern #01 with 150 μm filaments was further normalized to 100%.

**Fig. 10:**
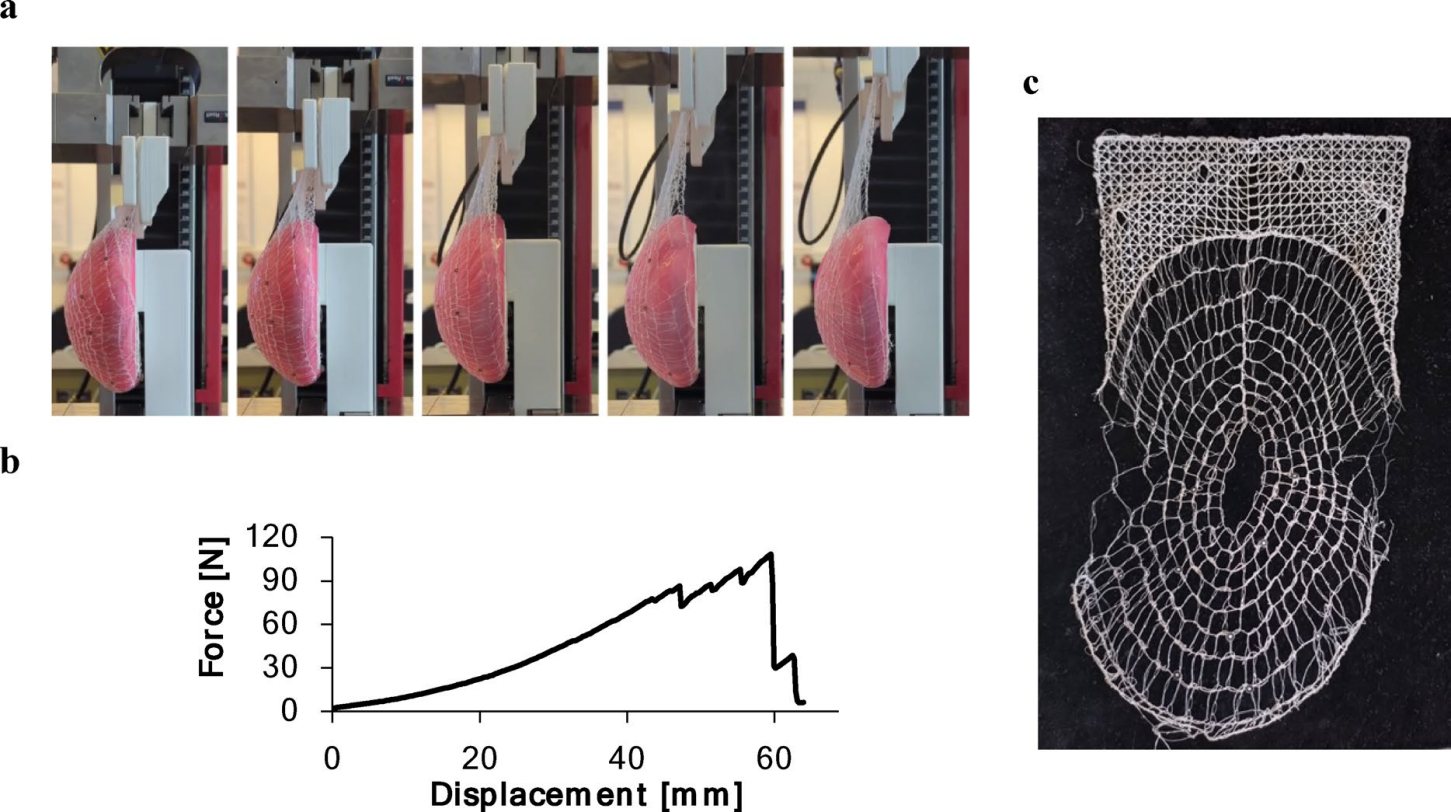
**a** Series of photographs showing a mesh pocket covering a silicone-coated 3D-printed implant model during tensile testing, from initial position (left) to failure (right); **b** Representative force– displacement curve; **c** Mesh after failure, displaying typical thread breakages on the left and right sides of the dome.

The stability of the mesh pockets was evaluated through tensile testing, determining the maximum force at failure (Fig. 10). The failure pattern was similar across pockets, with fractures occurring at the left and right sides of the dome.

The maximum force at failure depended significantly on filament diameter and pattern design (*p* < 0.001). The pockets with the standard pattern #01 were prepared using filaments of different diameters. As expected, the pockets made with thicker filaments withstood higher forces before breaking. The maximum force decreased from approximately 103 N for 150 μm filaments to 90 N for 125 μm filaments; however, this difference was not statistically significant. In contrast, significant reductions were observed for thinner filaments, dropping to 33 N for 67 μm filaments (*p* < 0.01) and 16 N for 49 μm filaments. The normalized tensile force did not change significantly for pattern #01 prepared with different filament diameters, indicating that the measured maximum force was mainly influenced by filament diameter and mesh mass, respectively.

**Fig. 11:**
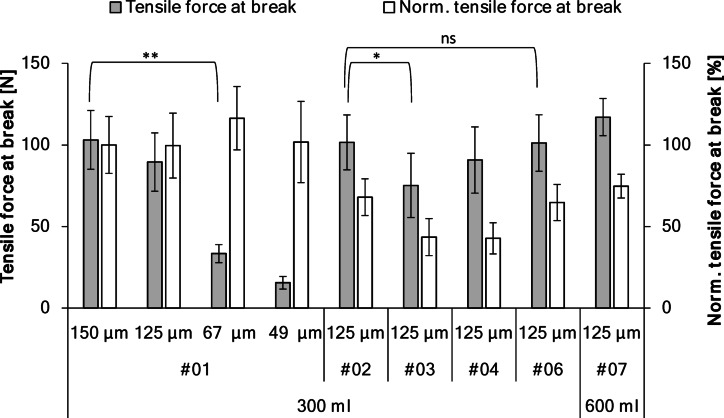
Overview of the prepared mesh pockets from tensile testing, showing the absolute maximum tensile force at break and the normalized tensile force at break (for comparison pattern #01 with 150 μm filament is set to 100%). Statistical significance was determined via one-way ANOVA with Tukey HSD post-hoc test. Brackets indicate specific pairwise comparisons of interest (** *p* < 0.01; ns = not significant).

Increasing the mesh density of the dome raised the maximum force from 90 N for pattern #01 to 102 N for pattern #02. Although numerically higher, this increase was not statistically significant. However, the normalized tensile force at break relative to the dome’s mass per unit area decreased, indicating that factors beyond mesh mass influence the tensile performance (Fig. 11).

Modifying the dome geometry significantly impacted mechanical stability. The pattern with the large central opening (#03) exhibited the lowest stability among the 125 μm variants (75 N), which was significantly lower than the dense dome pattern #02 (*p* < 0.01). The pattern with the flat upper segment (#04) exhibited an intermediate maximum force of approximately 91 N, which was not significantly different from the closed-dome patterns. In both cases, the normalized force at break was reduced compared to the closed-dome patterns (#01 and #02), indicating that a fully closed and well-fitting dome was crucial for achieving high maximum tensile force. The closed design evenly distributes stress, preventing high forces from concentrating at a single point and reducing the risk of filament breakage. The pocket with the completely flat top was not tested because the implant model fell out immediately when inserted.

Pattern #06, which was similar to pattern #02 but with a reinforced back, had a maximum force at break (101 N) that was statistically indistinguishable from its non-reinforced counterpart (102 N), confirming that the back reinforcement did not alter the failure load.The larger pocket, designed for a 600 ml implant, withstood a slightly higher maximum force of approximately 117 N, and its specific force also increased, compared to the corresponding 300 ml pocket (#02) from which it was derived, though this difference was not statistically significant.

**Fig. 12:**
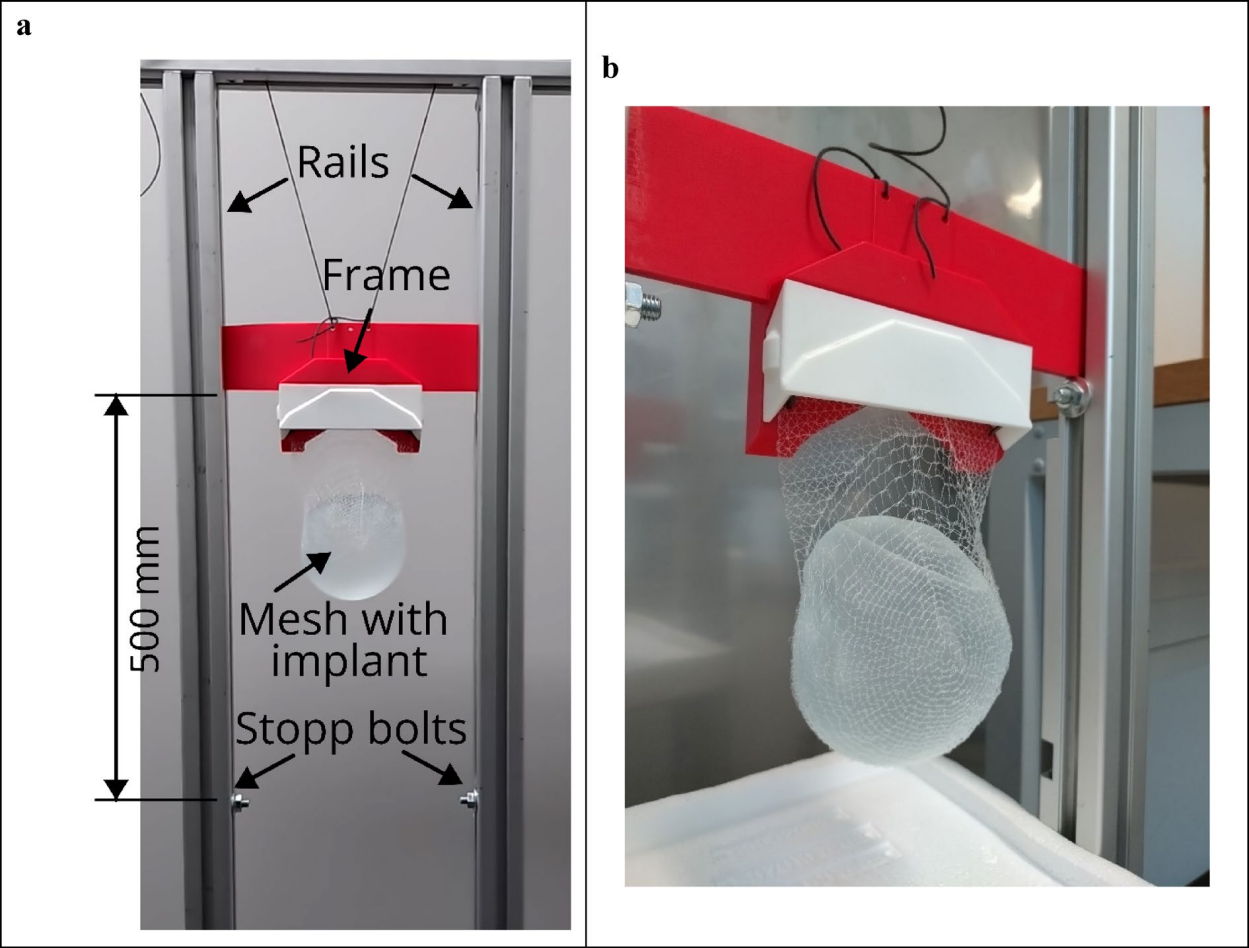
Drop test experimental setup: **a** the mesh pocket containing a silicone implant is suspended from a guided frame between vertical rails, shown in the starting position 500 mm above the stopping bolt; **b** close-up view of the assembly in the resting position immediately after impact, demonstrating successful containment of the implant without displacement.

Drop tests were performed on mesh pockets that were fabricated using a 125 μm filament (Fig. 12, Suppl. Video 01). The failure behavior observed in these tests differed significantly from that seen in quasi-static tensile tests (Table 1). All 300 ml mesh pockets with a dome kept the lightweight 3D-printed implant model in place during the drop test, with no displacement or breakage. However, when the heavier silicone implant was inserted, the thread on the back side of mesh pockets #01 and #02 broke, which was a type of mesh failure not observed in quasi-static tests. Mesh #03 featured a large opening in the dome segment that allowed the deformable silicone implant to squeeze through and fall out. This failure did not occur with the rigid 3D-printed model. For pattern #04, which had a flat segment instead of the dome opening, the silicone implant could not be held in place and fell out through the back upon impact (Suppl. Video 02). In contrast to pattern #02, pattern #06 with a reinforced back side, passed the drop test successfully with no displacement of the heavy silicone implant or breakage of the mesh (Suppl. Video 03).

**Table 1:** 500 mm drop test results for mesh pockets with 3D-printed (98 g) and silicone (277 g) implant models. A tick (✓) indicates that the implant was successfully secured, while other entries describe the observed failure mode.

	**3D-printed model implant (98 g)**	**Silicone implant (277 g)**
#01	✓	Back side thread failure
#02	✓	Back side thread failure
#03	✓	Fall out through front side
#04	✓	Fall out through back side
#05	Implant falls out to back side before drop test	
#06	✓	✓

### Finite element analysis

**Fig. 13:**
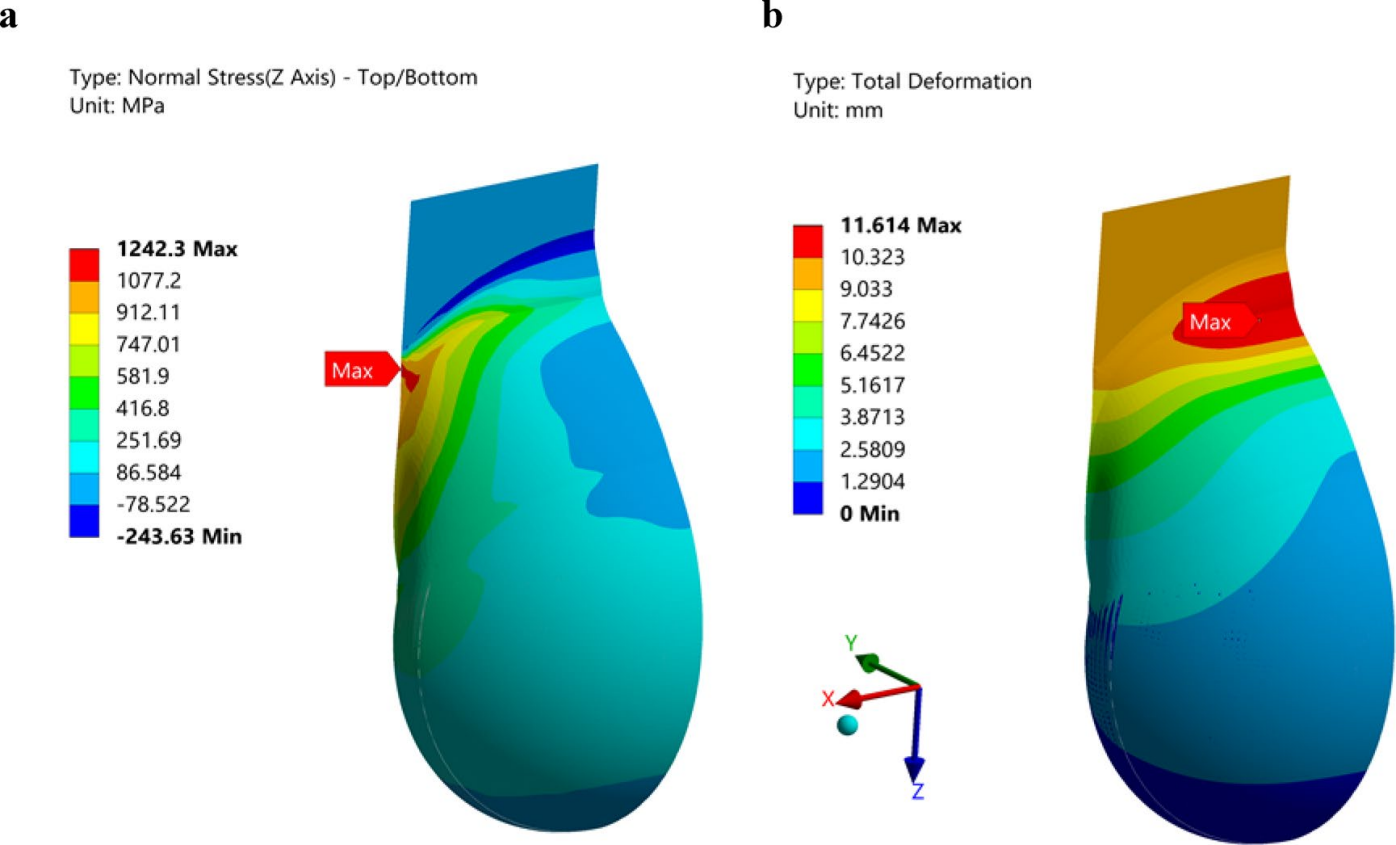
Finite element analysis results for **a** stress relative to Z axis and **b** total displacement.

FEA simulated uniaxial tensile loading to visualize stress distribution and identify regions of stress concentration. The simulation revealed a pronounced distribution of stress along the Z-axis (Fig. 13a) in the left region of the pocket, reaching 1242 MPa. While elevated stresses in this area were expected due to the combined effects of axial loading and lateral contraction, the sharp curvature transition between the dome and the attachment further intensified this effect. The displacement magnitude (Fig. 13b) indicated maximum deformation in regions where the pocket contracted along the Y-axis while simultaneously stretching in the Z-axis. The FEA results highlight the dome-attachment transition zone as a critical area for stress concentration, suggesting that smoother geometric transitions or localized reinforcements could reduce peak stresses and improve mechanical stability.

### 3D surface scanning

**Fig. 14:**
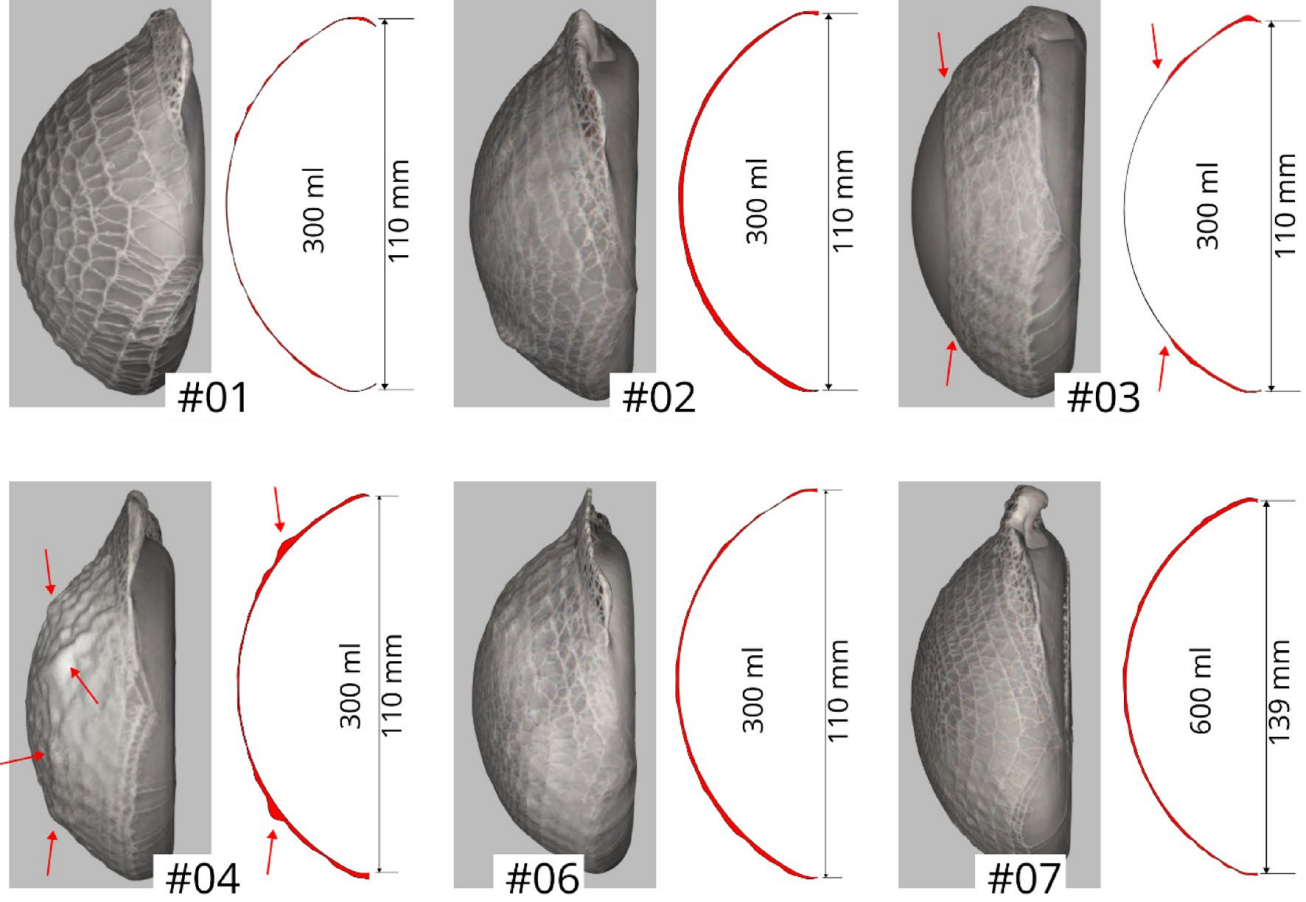
Cross-sectional analysis visualizing the gap between the mesh and implant surface. The space between the two contour lines (representing the implant and mesh edges) is filled in red to illustrate this gap. Sample **#01** exhibits minimal gap, indicating excellent conformity. Mesh **#02** shows a more pronounced visualized gap. For sample **#03**, the mesh edge and the large central opening are clearly distinguished; the red fill highlights the gap only within the meshed regions, contrasting with the open area. Red arrows at sample **#04** indicate observed wrinkles and folds, highlighting a significantly compromised fit. Meshes **#06** and **#07** show no pronounced wrinkles but exhibit visible gaps; **#06** has a gap size comparable to **#02**, and **#07** demonstrates good conformity across its larger volume.

The 3D surface scans (Suppl. Figure 2a) provided rapid, quantitative evidence of the mesh pockets’ fit around the implant models. By generating digital cross-sections (Suppl. Figure 2b) and visualizing the space between the mesh and implant (Suppl. Figure 2c), the analysis demonstrated the high conformity of the optimized 3D embroidery patterns (Fig. 14).

Pattern #01 achieved a near wrinkle-free fit with minimal elevation, demonstrating excellent adaptability to the curved surface. Patterns #02 and #06, which share the same dense dome geometry, exhibited a slightly larger gap distance compared to #01. This difference is likely due to the higher mesh density, which may have affected how the mesh adapts to the surface or how the scanner detects the mesh boundary. The scaled-up pocket (#07) also demonstrated good conformity across its larger volume.

For the open-dome pattern #03, the scan clearly distinguished the large central opening from the surrounding mesh. In the opening, the scan tracked the implant surface directly, while in the mesh-covered regions, a distinct offset was visible. In stark contrast, pattern #04, which incorporated a flat, non-drapable central segment instead of the opening of pattern #03, exhibited significant wrinkling and the largest observed gaps between the mesh and the implant surface. The reduced three-dimensional adaptability of this flat design confirms the limitations of conventional textile approaches when applied to complex curved geometries.

**Fig. 15:**
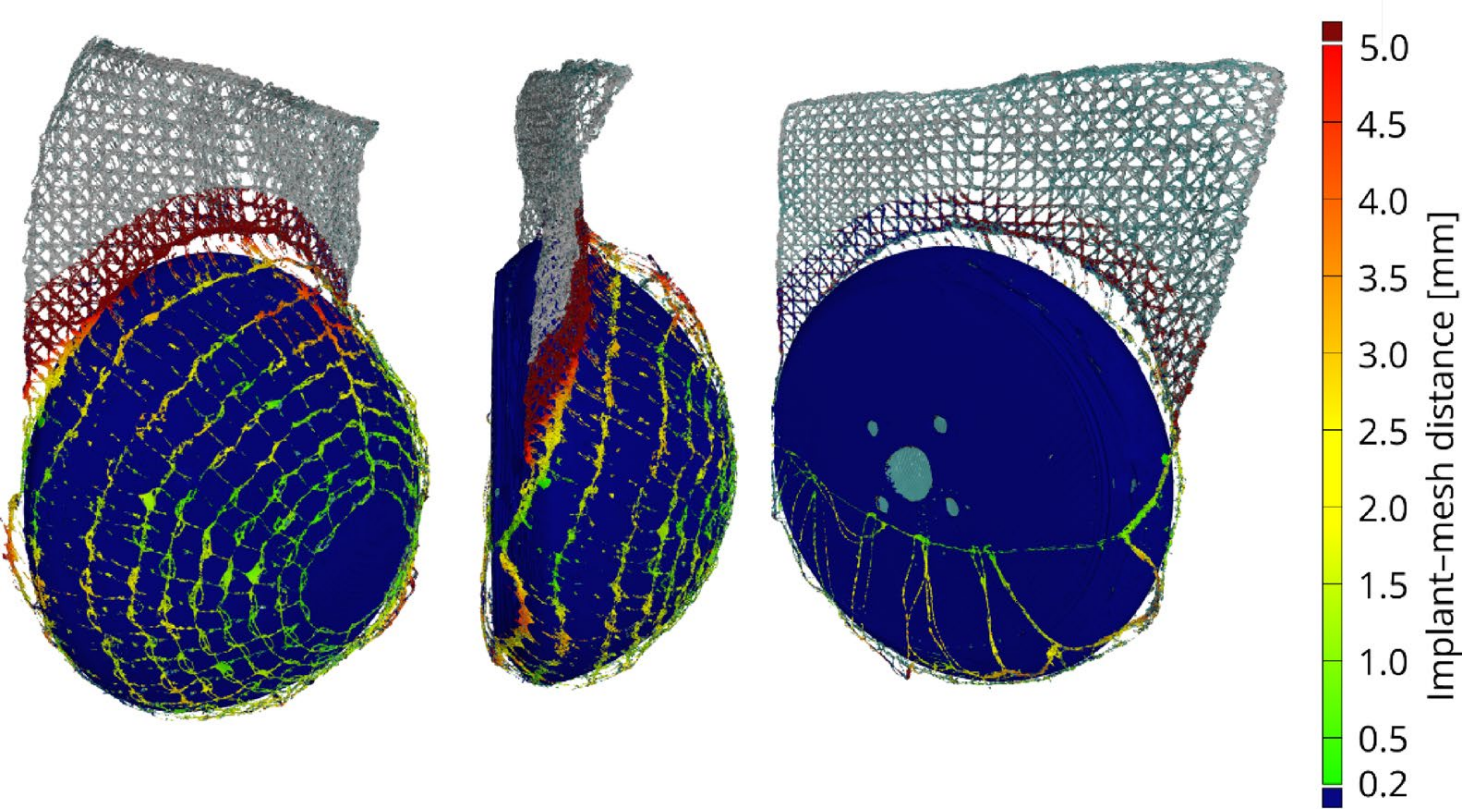
Results of the 3D surface scan of the embroidered mesh pocket on the implant model. The mesh is colorized using a distance map, where green indicates close conformity to the reference implant surface and red represents regions of greater deviation.

Although the second scanning setup is more time-consuming (approximately 5 h for a complete scan), it enables higher-resolution and more detailed visualization. The detailed scans confirmed that the mesh pocket #01 fit closely to the surface of the implant (Fig. 15). Using this method, the distance between the embroidered dome and the implant model could be measured and was mostly within the 1–2 mm range, indicating a tight fit. Only in a few localized areas were larger gaps observed, reaching up to approximately 5 mm. These results show that the embroidered mesh adapts well to the curved geometry of the implant, with only minor deviations in certain areas.

## Discussion

Commercial mesh pockets produced from flat textiles by cutting and sewing often display wrinkles, excess material, and suboptimal fit on curved implant surfaces due to the limitations of the conventional textile manufacturing approach. Machine embroidery, particularly in the production of free-standing lace, takes a fundamentally different approach to textile construction. Pairs of threads are sewn onto a removable base material to create structures based on pre-programmed patterns. Although this technique is less productive than weaving or warp-knitting, as only one pair of threads is processed at a time, it provides exceptional structural freedom. This allows for the design of highly tailored and complex patterns that cannot be achieved with conventional textile fabrication methods. This study demonstrates the versatility of embroidery in creating customized surgical meshes, especially for complex 3D textile meshes.

The design process was inspired by commercially available mesh pockets for breast implants and broken down into three distinct geometric segments: (1) a dome to form the front; (2) a back segment to hold the implant in place; and (3) an extension segment to enable anchorage to biological tissue. The geometry of the dome could be pre-calculated in order to estimate the final shape and fit of the implant. Deviations between the predicted and measured dome heights were attributed to filament bending and elastic restoring forces. These were corrected empirically, though future refinements should consider filament diameter and stitch density.

One significant advantage of embroidery-based designs is their scalability. For instance, a pocket designed for a 300 ml implant can be scaled up to 600 ml by proportionally increasing the number of concentric rings and meandering connections while preserving pore geometry and overall shape. This shows how adaptable the technique is for different implant sizes and how well it can be customized to suit each patient.

Pore geometry is a critical parameter for the clinical integration of meshes. Embroidery enables precisely defined pores because they are integral components of the programmed pattern. In this study, trapezoidal pores were fabricated, with median sizes of 4 mm. These pore sizes are within the clinically accepted range, promoting tissue integration while maintaining mechanical stability. The shape, size and distribution of the pores can be further adjusted by modifying the embroidery pattern.

The mass per unit area of the embroidered meshes (pattern #01: 12 g/m²) was notably lower than that of conventional mesh pockets (TiLOOP^®^ Bra Pocket: 16 g/m² and SERASYNTH^®^ MESH Pocket: 90 g/m²). Since embroidery allows threads to be placed precisely where needed, material usage is minimized and localized thickness variations (such as seam thickening in conventional mesh pockets) are avoided. This enables mesh structures that are significantly lighter than conventional knitted meshes, which are typically sewn into pockets from separate pieces overlapping each other. By reducing the mass per unit area, the total amount of foreign material implanted in the body is minimized (reduced material burden), which may reduce foreign-body immune response.

Mechanical testing confirmed the robustness of the embroidered mesh pockets. Dome-shaped pockets exhibited greater tensile force at break than flat or open-top variants, demonstrating the mechanical benefits of three-dimensional shaping. Drop tests revealed that targeted reinforcements, such as the addition of embroidered threads to the back segment, significantly enhanced stability. This demonstrates the ability of embroidery to be modified rapidly and precisely to enhance performance.

However, as this study focuses on manufacturing feasibility and structural characterization, a direct comparison with commercial products was omitted due to material differences and the lack of standardized testing protocols. Furthermore, due to the nature of embroidery, which joins material along stitching lines, it is not possible to produce fully enclosed or spherical structures such as the final mesh pocket directly. In this study, the back of the pocket was embroidered separately and folded to form a closed 3D shape, a process requiring manual intervention. However, future developments in process strategies or hybrid manufacturing approaches may overcome these constraints.

Free-standing lace embroidery provides a level of design freedom comparable to that of 3D printing or plotting. It relies solely on processed threads that can be produced with any standard embroidery machinery. The ability to predefine geometry, pore structure, and mass per unit area allows for a high degree of customization, making embroidery a promising technique for next-generation surgical meshes which combine a precise three-dimensional fit with material efficiency.

Future research on mesh pockets should include impact tests using high-speed cameras to analyze deformation and identify weak spots, thereby ensuring secure implant placement. Studies on degradable polymers and their degradation behavior are crucial for developing implants that remain in the body only until sufficient biological tissue has formed. Based on the geometric calculations presented here, reverse engineering could facilitate the creation of customized mesh designs for specific implants, enabling the rapid, on-demand production of personalized meshes for clinical use. Free-standing lace embroidery is a manufacturing technology for tailored textiles that is more generally underappreciated. It can produce virtually any textile structure with precise control over shape, fiber architecture, and integrated features, such as eyelets, reinforced areas, and multi-material designs, all with a single thread and an embroidery machine.

## Conclusions

This study demonstrates that free-standing lace embroidery is an innovative approach to producing 3D surgical meshes with near-net-shape accuracy. By digitally specifying the geometry and pore structure, the meshes achieve a precise fit, reduced material consumption, and a lightweight construction, providing distinct advantages over traditional cut-and-sew methods. Experimental results confirm that the embroidered meshes are mechanically robust and closely conform to implant surfaces with minimal wrinkling. Free-standing lace embroidery offers a flexible, scalable platform for developing patient-specific surgical meshes, providing more efficient, customizable solutions for breast reconstruction and has potential for other medical applications.

## Supplementary Information

Below is the link to the electronic supplementary material.

Supplementary Material 1

Supplementary Material 2

Supplementary Material 3

Supplementary Material 4

## Data Availability

The data that support the findings of this study are available from the corresponding author upon reasonable request.

## References

[1] Elschner, C. & Hahn, J. The Evolution of Embroidery Machines. In *Sewing Machines and Processes* (CRC, 2025). 10.1201/9781003293071-11

[2] Lin, R. et al. Digitally-embroidered liquid metal electronic textiles for wearable wireless systems. *Nat. Commun.* **13**, 2190. 10.1038/s41467-022-29859-4 (2022). 35449159 10.1038/s41467-022-29859-4 PMC9023486

[3] Goveia da Rocha, B., Tomico, O., Markopoulos, P. & Tetteroo, D. Crafting Research Products through Digital Machine Embroidery. In *Proc 2020 ACM Des Interact Syst Conf*., 341–50 (Association for Computing Machinery, New York, NY, USA, 2020). 10.1145/3357236.3395443

[4] Yang, Y., Chen, Y., Liu, Y. & Yin, R. Programmable and scalable embroidery textile resistive pressure sensors for integrated multifunctional smart wearable systems. *Adv. Fiber Mater.* **7**, 574–586. 10.1007/s42765-024-00506-5 (2025).

[5] Jiang, Y. et al. Shape-Changing Textiles with Machine Embroidery. In *Proc 37th Annu ACM Symp User Interface Softw Technol*., 1–15 (Association for Computing Machinery, New York, NY, USA, 2024). 10.1145/3654777.3676431

[6] Glazko, K. et al. Case Study of GAI for Generating Novel Images for Real-World Embroidery. arXiv (2025). 10.48550/arXiv.2510.16223

[7] Aishwariya, D. & Ramyabharathi, B. Lace through time: exploring history, types, materials, motifs, innovations and designing lace for non-textile products. *Man-Made Text. India*. **11**, 367–372 (2023).

[8] Gögele, C. et al. Co-Culture of mesenchymal stem cells and ligamentocytes on triphasic embroidered Poly(L-lactide-co-ε-caprolactone) and polylactic acid scaffolds for anterior cruciate ligament enthesis tissue engineering. *Int. J. Mol. Sci.* **24**, 6714. 10.3390/ijms24076714 (2023). 37047686 10.3390/ijms24076714 PMC10095212

[9] Wöltje, M. et al. Textile design of an intervertebral disc replacement device from silk yarn. *Biomimetics* **8**, 152. 10.3390/biomimetics8020152 (2023). 37092404 10.3390/biomimetics8020152 PMC10123607

[10] Jo, J., & Kao, C. H. L. SkinLace Freestanding Lace by Machine Embroidery for On-Skin Interface. In *Ext Abstr 2021 CHI Conf Hum Factors Comput Syst*., 1–6 (Association for Computing Machinery, New York, NY, USA, 2021). 10.1145/3411763.3451756

[11] Glazko, K., Portnova-Fahreeva, A., Mankoff-Dey, A., Psarra, A. & Mankoff, J. Shaping lace: Machine embroidered metamaterials. In *Proc 9th ACM Symp Comput Fabr*., 1–12 (Association for Computing Machinery, New York, NY, USA, 2024). 10.1145/3639473.3665792 10.1145/3639473.3665792 PMC12645458 41306447

[12] Kyosev, Y., Elschner, C., Bittrich, L. & Patel, P. Topology Based 3D Modelling of Textiles with Machine Embroidery Stitches, 1–6 (2024). 10.2478/9788367405355-001

[13] Baylón, K. et al. Present and future of surgical meshes: A review. *Membranes* **7**, 47. 10.3390/membranes7030047 (2017). 28829367 10.3390/membranes7030047 PMC5618132

[14] Ostapenko, E. et al. Prepectoral versus subpectoral Implant-Based breast reconstruction: A systemic review and meta-analysis. *Ann. Surg. Oncol.* **30**, 126–136. 10.1245/s10434-022-12567-0 (2023). 36245049 10.1245/s10434-022-12567-0 PMC9726796

[15] Li, Y., Xu, G., Yu, N., Huang, J. & Long, X. Prepectoral versus subpectoral Implant-Based breast reconstruction: A meta-analysis. *Ann. Plast. Surg.* **85**, 437. 10.1097/SAP.0000000000002190 (2020). 31913902 10.1097/SAP.0000000000002190

[16] Weinzierl, A., Schmauss, D., Brucato, D. & Harder, Y. Implant-Based breast reconstruction after Mastectomy, from the subpectoral to the prepectoral approach: an evidence-based change of mind?. *J. Clin. Med.* **11**, 3079. 10.3390/jcm11113079 (2022). 35683465 10.3390/jcm11113079 PMC9181810

[17] Chopra, S., Al-Ishaq, Z. & Vidya, R. The journey of prepectoral breast reconstruction through time. *World J. Plast. Surg.* **10**, 3–13. 10.29252/wjps.10.2.3 (2021). 34307092 10.29252/wjps.10.2.3 PMC8290458

[18] Becker, H. & Lind, J. G. The use of synthetic mesh in reconstructive, revision, and cosmetic breast surgery. *Aesthetic Plast. Surg.* **37**, 914–921. 10.1007/s00266-013-0171-8 (2013). 23860816 10.1007/s00266-013-0171-8 PMC3778996

[19] Kobraei, E. M., Cauley, R., Gadd, M., Austen, W. G. J. & Liao, E. C. Avoiding breast animation deformity with pectoralis-sparing subcutaneous direct-to-implant breast reconstruction. *Plast. Reconstr. Surg.* **4**, e708. 10.1097/GOX.0000000000000681 (2016). 10.1097/GOX.0000000000000681 PMC4995704 27579232

[20] Gfrerer, L. & Liao, E. C. Technique refinement in prepectoral implant breast reconstruction with Vicryl mesh pocket and acellular dermal matrix support. *Plast. Reconstr. Surg.* **6**, e1749. 10.1097/GOX.0000000000001749 (2018). 10.1097/GOX.0000000000001749 PMC5977955 29876185

[21] Vidya, R. & Iqbal, F. M. A guide to prepectoral breast reconstruction: A new dimension to Implant-based breast reconstruction. *Clin. Breast Cancer*. **17**, 266–271. 10.1016/j.clbc.2016.11.009 (2017). 28190760 10.1016/j.clbc.2016.11.009

[22] Casella, D. et al. TiLoop^®^ bra mesh used for immediate breast reconstruction: comparison of retropectoral and subcutaneous implant placement in a prospective single-institution series. *Eur. J. Plast. Surg.* **37**, 599–604. 10.1007/s00238-014-1001-1 (2014). 25339795 10.1007/s00238-014-1001-1 PMC4197328

[23] Grous, A. et al. Immediate breast reconstruction in breast cancer patients with the use of Serasynth and SeragynBR synthetic meshes: Single-oncological center experience, analysis of complications. *Pol. Przegl Chir.* **96**, 11–20. 10.5604/01.3001.0016.3172 (2023). 38629281 10.5604/01.3001.0016.3172

[24] Berna, G., Cawthorn, S. J., Papaccio, G. & Balestrieri, N. Evaluation of a novel breast reconstruction technique using the Braxon^®^ acellular dermal matrix: a new muscle-sparing breast reconstruction. *ANZ J. Surg.* **87**, 493–498. 10.1111/ans.12849 (2017). 25266930 10.1111/ans.12849

[25] Peng, H-T. Changes in Biomechanical properties during drop jumps of incremental height. *J. Strength. Cond Res.* **25**, 2510. 10.1519/JSC.0b013e318201bcb3 (2011). 21869631 10.1519/JSC.0b013e318201bcb3

