# Supplementary material for: Beyond decoration: free-standing lace embroidery for 3D shaped surgical mesh implants
Source: Sci Rep. 2026 Mar 4;16:8270. doi: 10.1038/s41598-026-36575-2 (PMC12966322; doi:10.1038/s41598-026-36575-2)
Supplement: Supplementary file 1 — Supplementary Material 1 [file 41598_2026_36575_MOESM1_ESM.docx]

| 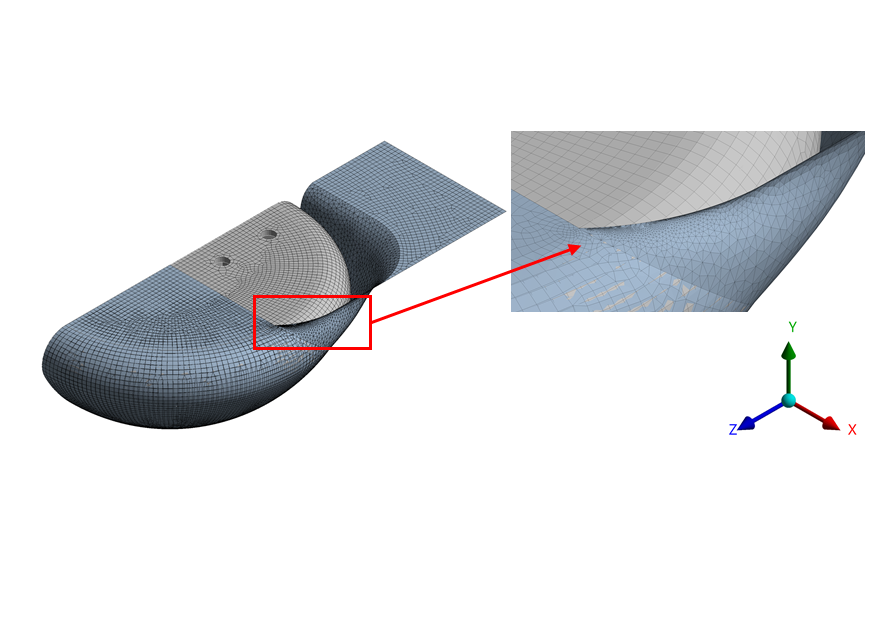 |
| --- |
| **Suppl. Fig. 1.** Mesh applied in the FEA model, highlighting the region with refined elements. |

| a  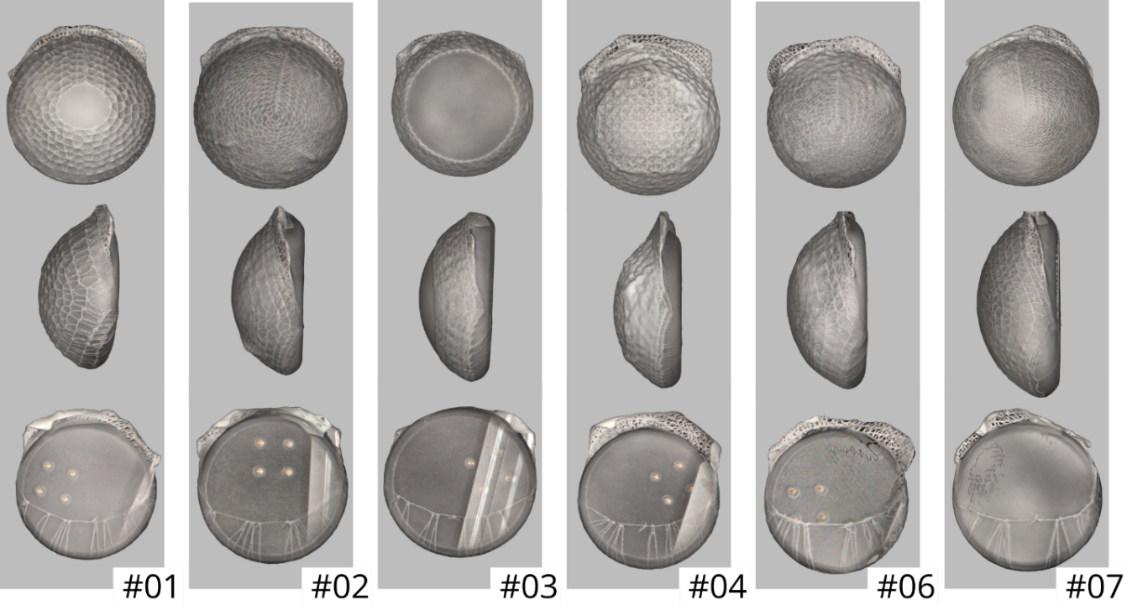 | |
| --- | --- |
| b  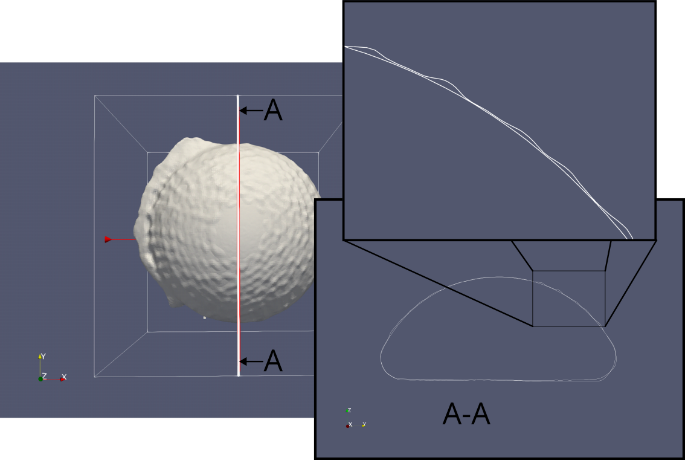 | c  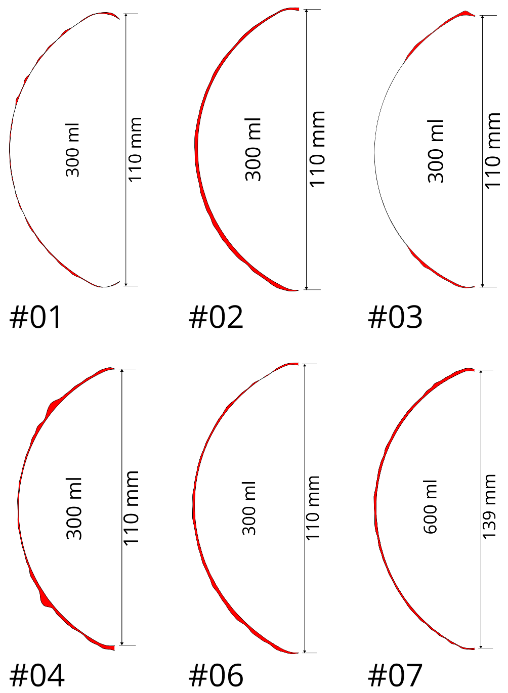 |
| **Suppl. Fig. 2.** **a** Rapid surface scans of various embroidered mesh pockets containing implant models, shown with applied photographic textures; **b** visualization of the plane used to extract a cross-section from the 3D scan, showing the two contour lines corresponding to the implant surface and mesh position; and **c** overview of the resulting contour lines from the cross-section, with the space between the two lines filled in red to illustrate the gap between the mesh and implant surface. | |


$r_{a,i}$ $s_{i}$ $g$ $a_{i}$ $c_{i}$ $\delta_{i}$ $\alpha_{i}$ ${\Delta h}_{i}$ $w_{a,i}$
